# Bexarotene leads to durable improvements in visual evoked potential latency: A follow-up study of the Cambridge Centre for Myelin Repair One trial

**DOI:** 10.1177/13524585241233177

**Published:** 2024-03-01

**Authors:** Christopher E McMurran, Trisha Mukherjee, J William L Brown, Alasdair J Coles, Nick G Cunniffe

**Affiliations:** Department of Clinical Neurosciences, University of Cambridge, Cambridge, UK; Department of Clinical Neurosciences, University of Cambridge, Cambridge, UK; Department of Clinical Neurosciences, University of Cambridge, Cambridge. UK/NMR Research Unit, Queen Square Multiple Sclerosis Centre, University College London (UCL) Institute of Neurology, London. UK/Clinical Outcomes Research (CORe) Unit, The University of Melbourne, Melbourne, VIC, Australia; Department of Clinical Neurosciences, University of Cambridge, Cambridge, UK; Department of Clinical Neurosciences, University of Cambridge, Cambridge, UK

**Keywords:** Multiple sclerosis, remyelination, clinical trial, evoked potentials, visual, bexarotene, optic neuritis

## Abstract

The Cambridge Centre for Myelin Repair One (CCMR-One) trial showed that 6 months of bexarotene reduces visual evoked potential (VEP) latency in people with relapsing-remitting multiple sclerosis (MS). In a single-centre follow-up study of these participants, we re-examined full-field VEP and clinical assessments. Twenty participants (12 bexarotene and 8 placebo) were seen on average 27 months after their trial involvement. In an analysis of all eyes with recordable signal (24 bexarotene and 14 placebo), the adjusted bexarotene-placebo treatment difference in P100 latency was −7.79 (95% confidence interval (CI) = −14.76, −0.82) ms, *p* = 0.044. We conclude that there were durable improvements in VEP latency, suggesting long-term benefits from exposure to a remyelinating drug.

## Introduction

The Cambridge Centre for Myelin Repair One (CCMR One) trial of bexarotene^1^ was the third phase 2 trial – after the ReBUILD study of clemastine,^2^ and the RENEW trial of opicinumab^3^ – to show a remyelinating effect through a reduction in the latency of the visual evoked potential (VEP).^4^ Unfortunately, 300 mg/m^2^ bexarotene was poorly tolerated, but the lessons learned – the need for selective retinoid X receptor gamma (RXR-γ) agonists and informing future remyelination trial design – may prove its legacy.^1,5^

In the present work, we conducted a follow-up study of the original trial participants of CCMR One at one of the two study sites to investigate the long-term effects of bexarotene. We tested the hypothesis that the improvements in the full-field VEP (FF-VEP) latency that were observed in the original trial were durable, which might indicate lasting effects from treatment with a remyelinating drug.

## Patients and methods

The CCMR One was a randomised, double-blind, placebo-controlled, parallel-group, phase 2 study conducted in Cambridge and Edinburgh (ISRCTN 14265371).^1^ In this follow-up study, participants were recruited who had been in the CCMR One trial in Cambridge. The study was approved by Health and Care Research Wales (HCRW) Ethics Committee (20/WA/0294) and undertaken in accordance with Good Clinical Practice. All participants gave written informed consent.

FF-VEPs were assessed using a VisionSearch Plus (VisionSearch, Sydney, NSW, Australia); the FF-VEPs undertaken during the CCMR One trial had been performed on a Nicolet Synergy system (Optima Medical Ltd, UK). In both instances, FF-VEPs were elicited by a 2-Hz reversing check pattern of size of 60 minutes of arc with signal recorded from a channel formed between gold-cup electrodes positioned frontally in the midline and 2.5 cm above the inion (Fz-Oz). Between 3 and 5 recordings were taken per eye and the weighted average used to measure P100 latency. All participants had a repeated Expanded Disability Status Scale (EDSS) assessment.^6^

Statistical analysis was performed using R (Version 1.3.1093). Treatment effects between the follow-up visit and the original trial were tested using linear mixed models for eyes nested within patients, with patient random intercepts, regressing the change in P100 latency on treatment group and the baseline P100 latency, with the trial minimisation factors (age (⩽40/>40 years), gender and EDSS (⩽4.0/>4.0)) as covariates. For EDSS, a corresponding multiple regression on a group indicator, with the aforementioned age and gender covariates, was used. This was further validated using a non-parametric bias-corrected and accelerated bootstrap with 1000 replicates.

## Results

Between 10 December 2020 and 6 April 2021, 20 out of the 31 CCMR One participants from Cambridge consented to participate (Table 1). Recruitment occurred during the COVID-19 pandemic, so many expressed reluctances to attend a healthcare setting because of infection risk. Participants were seen on average 27 (standard deviation (SD) = 4.5) months after their original trial participation. Clinical relapses in two participants, radiological activity in one and lymphopaenia in one further participant had led to treatment escalation from dimethyl fumarate. Two participants had since been recently diagnosed with secondary progressive MS; though both remained on dimethyl fumarate at the time of enrolment. No participants had had an episode of acute optic neuritis since their participation in CCMR One.

**Table 1.** Baseline variables of those who attended the CCMR One follow-up visit.

	Bexarotene	Placebo
Total number of participants (number eligible from original trial)	12 (16)	8 (15)
Number converted to secondary progressive MS	1	1
Age, years; mean (SD)	44.3 (6.3)	42.8 (4.8)
Sex		
Female	7	3
Male	5	5
Disease duration, years; mean (SD)	11 (5.9)	8.4 (5.8)
Number of patients with clinical relapses since CCMR One	1	1
Disease-modifying drug		
Dimethyl fumarate	9	7
Cladribine	2	1
Fingolimod	1	0
Total number of VEP recordings with sufficient quality for inclusion (number of eyes)	24	15^a^
Baseline P100 latency, ms; mean (SD)	132.3 (17.7)	126.1 (22.3)

SD: standard deviation; CCMR: Cambridge Centre for Myelin Repair Trial Number One; VEP: visual evoked potential.

Data are presented by trial group.

a One eye included in this group had an unrecordable P100 latency at the baseline visit of CCMR One.

**Figure 1.**
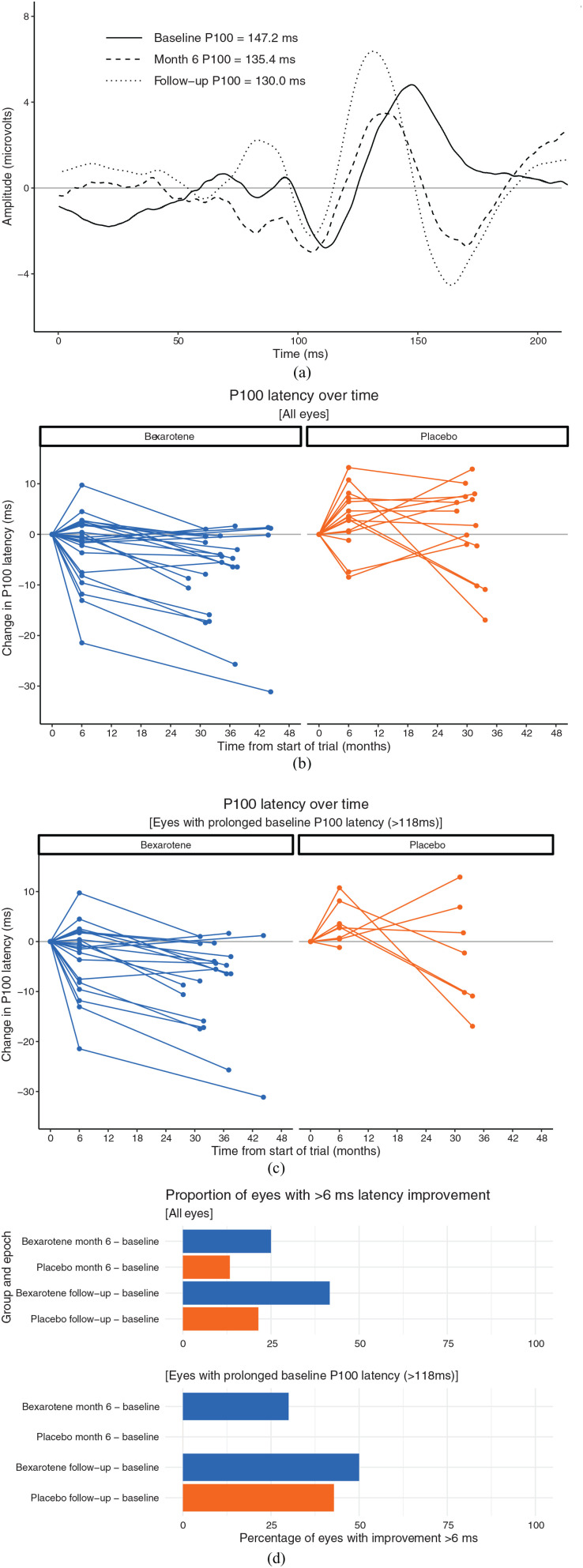
The change in full-field P100 latency over time. (a) Representative averaged VEP signals from the left eye of a participant across the three assessments: baseline and month 6 of the CCMR One trial, and the follow-up visit 2 years later. This participant had no history of clinical optic neuritis and had been randomised to bexarotene in the original trial. (b) and (c) The change in P100 latency for all eyes (b) and for just those eyes with a baseline P100 latency >118 ms (c) over course of the baseline and 6-month visits of CCMR One, and the follow-up assessment, divided by treatment group. (d) The percentage of eyes with more than 6 ms improvement in latency delay.

Thirty-eight out of 40 FF-VEP recordings were of sufficient quality to be analysed at both the CCMR One baseline and the follow-up visit recordings (Figure 1(a)). With all eyes included (24 bexarotene and 14 placebo), there was a statistically significant difference between the follow-up and baseline P100 latencies of the two trial arms: the adjusted treatment difference was −7.79 (95% confidence interval (CI) = −14.76, −0.82) ms, *p* = 0.044 (Figure 1(b)). When only eyes with a baseline P100 latency > 118 ms were included (20 bexarotene and 7 placebo), the trend to improvement in P100 VEP latency remained but was not statistically significant: the adjusted treatment difference was −5.39 (95% CI = −16.11, 5.32) ms, *p* = 0.343 (Figure 1(c)). In post hoc analyses, bexarotene treatment was associated with higher proportions of eyes with significant latency improvements (considered as a reduction of ⩾6 ms (Green, Gelfand and Cree, 2017)) compared to placebo over the 6-month trial period, and between the follow-up visit and original trial baseline (Figure 1(d)).

There was no treatment difference between the two groups on EDSS: the difference, adjusted for age and gender, was −0.31 (95% CI = −1.37, 0.74), *p* = 0.569. In a sensitivity analysis – excluding participants that had clinical relapses or radiological activity after trial participation – the EDSS difference was −0.25 (95% CI = −1.32, 0.81), *p* = 0.644.

## Discussion

This follow-up study to CCMR One has shown that, in this group of participants, the FF-VEP latency improvements observed in the original trial were durable. These data suggest that bexarotene has a remyelinating effect in humans, sustained years after the treatment period has concluded.

There are limitations to consider. First is the small number who agreed to participate, taken from only one of the trial sites, introducing the possibility of selection bias. Second, the change in those eyes with a baseline P100 latency of >118 ms was not statistically significant despite a larger treatment effect size relative to the CCMR One trial (−5.39 ms vs −4.06 ms); this might reflect the small numbers in the placebo group (7 eyes). Third, a potential confounding factor is that the follow-up clinic used a VS+ device, in comparison to a synergy (Optima medical) set-up during the trial.

Durability of VEP latency changes after exposure to a putative remyelination drug has been suggested before. In the ReBUILD study of clemastine, sustained VEP improvements were observed 2 months after clemastine discontinuation in one of the trial groups.^2^ In addition, in the RENEW trial of opicinumab, the VEP was repeated 8 weeks after IMP discontinuation, at which point the treatment difference in the per-protocol sample had increased to -9.1 ms, from −7.6 ms at the end of the 24-week treatment period.^3^ Finally, the RENEWED 2- to 3-year follow-up sub-study of 52/82 of the original RENEW trial participants reported a full-field VEP latency recovery between the opicinumab and placebo groups of −6.0 (95% CI = −14.6, 2.6) ms (*p* = 0.17) in the per-protocol sample.^7^

This sub-study of CCMR One is, to our knowledge, the second conducted at a time years remote from participation in a remyelination trial and the first to return a statistically significant result over this time scale. We conclude that this supports the increasingly clear position that pharmacological promotion of remyelination in people living with multiple sclerosis is possible and indicates a sustainability to repair following treatment with a remyelinating drug.
